# Perceptions and experiences of young Black South African women with obesity from a low socioeconomic community after following a 12-week structured exercise intervention

**DOI:** 10.3389/fspor.2022.813339

**Published:** 2022-10-05

**Authors:** Lindokuhle P. Phiri, Lisa K. Micklesfield, Amy E. Mendham, Julia H. Goedecke, Anniza de Villiers

**Affiliations:** ^1^Health Through Physical Activity, Lifestyle and Sport Research Centre (HPALS), FIMS International Collaborating Centre of Sports Medicine, Division of Physiological Sciences, Department of Human Biology, Faculty of Health Sciences, University of Cape Town, Cape Town, South Africa; ^2^SAMRC/WITS Developmental Pathways for Health Research Unit (DPHRU), Department of Paediatrics, School of Clinical Medicine, University of the Witwatersrand, Johannesburg, South Africa; ^3^Biomedical Research and Innovation Platform, South African Medical Research Council, Tygerberg, South Africa; ^4^Non-communicable Diseases Research Unit, South African Medical Research Council, Tygerberg, South Africa

**Keywords:** exercise, training environment, barriers and facilitative factors, self-efficacy, weight loss

## Abstract

**Background:**

Previous research has shown that Black South African (SA) women perceive a bigger body size to be acceptable and desirable, but nonetheless have shown interest in participating in community-based exercise programmes. This study aimed to investigate perceptions and experiences of participating in a 12-week exercise intervention designed to study the mechanisms of insulin sensitivity and secretion in young Black SA women with obesity.

**Methods:**

Qualitative data was collected from young (23 ± 2.9 years) Black SA women (*n* = 17) residing in a low-income setting in Cape Town, who took part in a 12-week structured exercise intervention. Focus group discussions (FGDs) and in-depth interviews (IDIs) were conducted 1–4 months after the completion of the intervention. These were all audio recorded and took between 45 and 60 min. The recordings were transcribed, translated and qualitative content analysis, entailing a systematic process of coding and identification of salient themes, was conducted using the ATLAS.ti software.

**Results:**

Six broad themes were identified from participants' experiences and perceptions: motivational factors, acceptability of the programme, barriers, sustainability and influencing others, benefits of being physically active, definitions and perceptions of exercise. Anticipated weight loss and financial remuneration were identified as motivational factors for enrolment and retention in the exercise programme. Aspects of the training environment and feelings of wellness appeared in the acceptability, sustainability and benefits themes, whereas time scheduling and travel constraints were regarded as barriers. Exercise was perceived as the maintenance of a healthy body, and in some cases, only relevant for specific groups.

**Conclusion:**

Financial considerations played an important role in participants enrolling and staying in the 12-week exercise intervention. Participants liked many aspects of the intervention and identified physical and mental benefits that seemingly outweighed the barriers and disliked aspects of the programme. Optimizing the acceptability of exercise programmes and maximizing the opportunity for participants to experience improved mental well-being may contribute to attracting and retaining young Black SA women in exercise programmes.

## Introduction

Participating in regular physical activity has been consistently linked with improved physical and mental well-being (1) and has been shown to play an important role in obesity management (2). Studies have shown black South Africa (SA) women prefer a bigger body size as it is associated with beauty, dignity, confidence (3), respect from others, happiness and wealth (4, 5), with some women commenting that “Our culture says that you are supposed to be fat” (6). African women, therefore have historically not perceived obesity as a threat to their health (7) and on the contrary have been reported to perceive thinness to be associated with diseases such as Human Immunodeficiency Virus (HIV), Acquired Immune Deficiency Syndrome (AIDS) and tuberculosis (TB) (8, 9).

More recently a transition in the perception of obesity toward Western norms of thinness in young SA women have been reported (10, 11). However, the latest South African Demographic and Health Survey showed that ~67% of Black SA women are overweight or obese (12), which has occurred alongside reduced physical activity and an increase in the prevalence of non-communicable diseases (NCDs) (13). Accordingly, exercise interventions designed to alter physical activity patterns and prevent the development of NCDs in young women with obesity are warranted. Findings of previous qualitative work in our study community reported that young Black SA women from a low-income setting were interested in participating in community-based exercise interventions that incorporate dancing, aerobics and lifting weights as a strategy to lose weight and improve health (6).

However, there are barriers to engaging in physical activity in people living in resource-constrained environments, which include lack of time, cost, accessibility to facilities, other people's perceptions and unsafe residential environments (14). However, little is known about how these barriers conspire to affect the adherence and sustainability to a structured exercise programme. It is anticipated that exploring women's perceptions and experiences of “exercise” after participating in an exercise intervention will assist in identifying barriers, enablers and goals that can be used to design future long-term exercise interventions. Accordingly, this study aimed to investigate the perceptions and experiences of a 12-week structured exercise intervention in a group of young Black SA women with obesity from a low-resource setting.

## Materials and methods

### Setting and exercise intervention

This qualitative study includes a sub-sample of women who participated in the primary study that examined the mechanisms underlying changes in insulin sensitivity and secretion in response to a 12-week exercise intervention in young Black SA women with obesity (15). The detailed protocol, trial registration (PACTR201711002789113), methods, consort diagram and baseline characteristics of the participants in the primary study have been previously published (15). The study was approved by the Human Research Ethics Committee at the University of Cape Town (HREC REF: 054/2015). This study was performed in accordance with the principles of the Declarations of Helsinki (1964, amended last in the Fortaleza Brazil, 2013) International Conference on Harmonization (ICH) Good Clinical Practice (GCP), and the laws of South Africa. Participants provided written informed consent prior to screening and testing procedures.

The first author was a PhD fellow at the time of the study. She has experience in qualitative research and is an exercise specialist with an interest in community-based exercise interventions.

### Participants and intervention design

The participants were a convenience sample of Xhosa-speaking women recruited from a low-socioeconomic area in Cape Town, SA. Based on the 2015 City of Cape Town socioeconomic profile, low-income areas are classified using average household income (16). The selected area is classified as a low socioeconomic area, with only 31% of the participants earning more than 5,000 South African Rands (ZAR), equivalent to ~290 United States Dollars ($) a month (16).

Participant recruitment ensured the following inclusion criteria: (i) South African women self-reported Black (both parents isiXhosa) and between the ages of 20–35 years; (ii) no surgical procedures within the last 6 months; (iii) weight stable (weight not changed more than 5 kg or no change in clothes size over the past 6 months); (iv) physically inactive (self-reported < 60 min a week of regular physical activity and/or planned moderate to vigorous intensity physical activity); (v) on injectable contraceptive (depot medroxyprogesterone acetate, 400 milligram (mg); (vi) no known metabolic or inflammatory diseases; (vii) no hypertension, diabetes, HIV or anemia; (viii) not taking any medications; (ix) non-smokers; (x) not currently pregnant or lactating; (xi) no orthopedic or medical problems that may prevent exercise participation; (xii) obese (body mass index (BMI) between 30 and 40 kg/m^2^. At baseline, BMI was measured and socio-demographic questionnaires, including age and measures of education and employment status, were completed by the participants with assistance of an isiXhosa-speaking research assistant.

Forty-five women met the inclusion criteria and were randomized into either an exercise group (*n* = 23) who participated in supervised aerobic and resistance exercise training; 40–60 min, 4 days per week, or a control group (*n* = 22) who were asked to maintain their normal physical activity and dietary behaviors. The project manager conducted block randomization after the participants completed the pre-intervention screening to ensure that researchers performing the testing were blinded to group allocation. A final sample of 35 participants completed the intervention (*n* = 20 in the exercise group; *n* = 15 in the control group).

Both groups were encouraged to maintain their normal dietary patterns. The exercise sessions were prescribed and included both aerobic and resistance training, and were conducted in a group setting and supervised by an exercise specialist registered with the Health Professions Council of South Africa (HPCSA). Participants in the control group were provided the opportunity to participate in the exercise sessions following the completion of post-intervention testing. Eight control group participants opted to join the exercise sessions. Heart Rate monitors (A300, Vantage NV, Polar, Finland) were used to monitor heart rate (HR) during every exercise session. The aerobic training component included dancing, running, skipping, and stepping at 75–85% of peak HR. The resistance exercises started with participants using their own body weight to perform exercises such as squats, lunges, and push-ups. Participants then progressed to using equipment such as elasticated bands and weights. Both aerobic and resistance exercises were altered to ensure progression and to maintain the required intensity throughout the intervention. Participants received a total of 3,800 ZAR over the course of the study which covered 30 ZAR per day to cover their transport costs to the training venue (4 × training sessions per week for 12 weeks, plus 3 monthly monitoring visits). Furthermore, to compensate the participants for their time and inconvenience, they were reimbursed 20 ZAR per hour for each training session and 50 ZAR per hour for the testing sessions (total of 17 h of testing and monitoring). The control group were similarly reimbursed for the testing sessions, and if they chose to participate in the exercise sessions post-intervention, they were similarly compensated.

### Post-intervention qualitative data collection and analysis

Focus group discussions (FGDs) and in-depth interviews (IDIs) were conducted in 2017, 1–4 months after the completion of the 12-week intervention to ensure that there were enough participants for each group discussion. All recruited participants (*n* = 45) were invited telephonically to participate in the FGDs. Participants who dropped out of the intervention (*n* = 10) and participants in the control group who rejected the opportunity to participate in the intervention at the end of the study (*n* = 7), were also contacted, with the intention to understand potential barriers relating to participating in the intervention. However, we were either unsuccessful in contacting these participants or they were not willing to participate. During the telephone recruitment for the FGDs, participants were informed that they would be remunerated with R50 ZAR to compensate for their transport, time, and inconvenience to participate in the FGD. Two of the eight participants in the control group who completed the post-intervention exercise training participated in the interviews. Participants who seemed to have the strongest opinions during the FGDs were invited to participate in the IDIs, optimizing the opportunity to further explore the themes identified and adding to the richness of the information collected during the FGD (17). The moderator and the scribe selected these participants in a debriefing session after each FGD, using the notes taken by the scribe to consider the flow of the group discussion and the contribution of the various participants.

The semi-structured focus group questions were formulated by the research group and were based on previous qualitative work done in a similar group of women from the study community (6). Following the question formulation, we sought independent input from external researchers with extensive experience in working with women from similar settings. The focus group questions were aimed at investigating the participants' perceptions about the exercise intervention, and barriers and motivators that might have influenced compliance during the exercise intervention (Table 1). The IDIs contained a single open-ended question. The interviewer allowed the participant to share her experiences of the exercise sessions (Table 1), while prompting her to elaborate on salient points arising from the focus groups. The purpose of the IDIs was to eliminate the influence of others' opinions that could have occurred during the FGDs (18).

**Table 1:** Focus group discussion and in-depth interview question schedules.

**Number**	**Interview schedules**
	**Focus group discussion questions**
1	Can you please briefly share with us what you have been up to since we last saw you?
2	What is the first thing that comes to mind when you hear the word “exercise”?
3	What are your perceptions about exercise?
4	What are some of the things that influenced you to attend the exercise sessions? This can either be barriers or motivating factors.
5	What did you like the most about the exercise sessions?
6	What did you not like about the exercise sessions?
7	How did the exercise training affect your daily life?
8	What did your friends and family have to say when they found out that you are exercising?
9	Is there anything that could be changed about the exercise training sessions to encourage you to continue exercising and for your friends and family to start exercising?
10	Of all the questions that were discussed today, is there one which you feel was the most important to you?
	**In-depth interview question**
1	How did you experience the exercise sessions?

A trained facilitator and scribe who were fluent in both English and IsiXhosa, the vernacular of the study population, conducted all the FGDs and IDIs. This ensured that no participant was excluded from the FGD because of language barriers. At the start of each FGD the facilitator and scribe introduced themselves and explained the purpose and reasons for doing the research. The FGDs and interviews were audio recorded and a scribe made notes during the FGDs. The FGDs and IDIs were conducted at a venue at the university and took between 45 and 60 min. No time restriction was set for these discussions and the duration was deemed appropriate for the number of participants in the FGDs and the question schedules. The audio recordings were transcribed from IsiXhosa into English by an independent translator, fluent in both English and IsiXhosa. The accuracy of the transcriptions both in tone and language were checked against the notes taken by the scribe by the first author.

The first author (LP) applied qualitative content analysis to analyse the transcripts (19) using the Atlas.ti Qualitative Data Analysis Software (Scientific Software Development GmbH, Berlin, Germany). This methodological approach allowed description and interpretation of the data using quantification of the data to develop themes. The analysis process started with reading the transcribed data in detail. Thereafter, a consensus process of drafting a coding framework was followed with another researcher (AD) and the codes were then systematically applied to the transcripts. If relevant, applicable codes were applied more than once to the data generated from the same participant. Main themes and sub-themes were then generated by LP and reviewed by both LP and AD after which clear definitions and names for each theme and subtheme were generated. These were checked by the rest of the research group and suggested changes were incorporated in the final document. Data saturation was achieved in the sense that all intervention and control participants, and all dropouts, were invited to participate in the qualitative study and all those who agreed, took part in the FGDs. The voices of most of the participants were therefore heard and additional data was collected through the IDIs to ensure that all issues raised in the FGDs were adequately explored.

## Results

Descriptive characteristics of the participants have been described elsewhere (20). The women were all of isiXhosa ancestry and from a low-income community. From all the contacted participants, 17 participants agreed to participate in the FGDs (Exercise, *n* = 15; Control, *n* = 2). There were no differences in age (24 ± 4 vs. 24 ± 4 years), BMI (33.2 ± 2.4 vs. 34.3 ± 3.0 kg/m^2^) or socio-demographic characteristics, including education (7 vs. 5 participants with tertiary education), household income (75 vs. 72% earned >R5000/month) or housing density (1.3 ± 0.6 vs. 1.4 ± 0.9 people living in the household/room, between the participants who agreed to participate in the FGD and those who did not, respectively.

**Figure 1:**
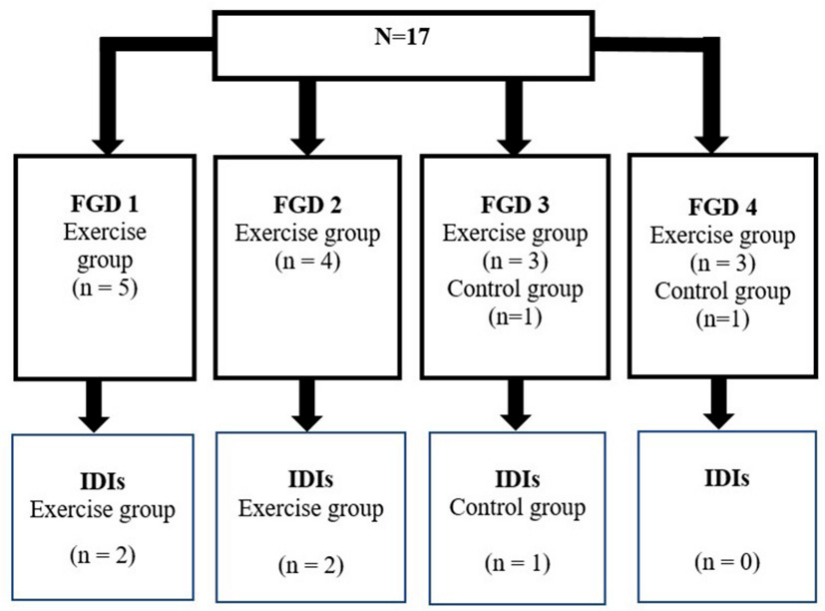
Participant distribution for the focus groups discussions (FGD) and in-depth interviews (IDI).

Five of the FGD participants were selected (Exercise, *n* = 4; Control *n* = 1) to complete IDIs. Two FGDs (FGD 1 and 2) comprised of only exercise participants who completed the intervention, and the other two FGDs (FGD 3 and 4) included a mix of participants from the exercise and control groups. Each FGD consisted of between three to five participants (Figure 1).

### Perceptions of the exercise intervention and the perceived barriers and facilitators

Six themes (T) with 2–3 subthemes (ST) each were identified during the analysis process. These themes and subthemes are presented in Table 2. Numbers included in the table reflect the number of fragments of experiences and perceptions brought together to first form the subthemes and later the main themes.

**Table 2:** Experiences and perceptions after a 12-week structured exercise intervention.

**Number**	**Theme**	**Subthemes**	**Number of quotations coded**
1	Motivational factors	Extrinsic (financial, weight loss, training environment) and intrinsic (enjoyment and mental wellness) motivation to continue with the intervention	44
		Extrinsic motivation to enroll in the intervention (financial and anticipated weight loss)	27
		**Total quotations**	**71**
2	Exercise program acceptability	Aspects of wellness and the training environment participants liked	28
		Aspects of the training environment and exercise regime that participants disliked	26
		**Total quotations**	**54**
3	Barriers	Travel constraints	11
		Time	10
		**Total quotations**	**21**
4	Sustainability and influencing others	Serve as influencer	16
		Keep on exercising	4
		**Total quotations**	**20**
5	Benefits of being physically active	Improved self-efficacy	7
		Disease prevention and reduced stress levels	5
		Lifestyle changes	4
		**Total quotations**	**16**
6	Definitions and perceptions of “exercise”	Maintaining a healthy body	6
		Change in body composition	3
		Fit and active	1
		**Total quotations**	**10**

Table 2 shows that experiences relating to motivation and the acceptability of the intervention were the themes with the most quotations attached to them. These were followed by barriers experienced; sustainability and being an influencer; the perceived and experienced benefits of exercise, and lastly how the term “exercise” was perceived and defined.

**Figure 2:**
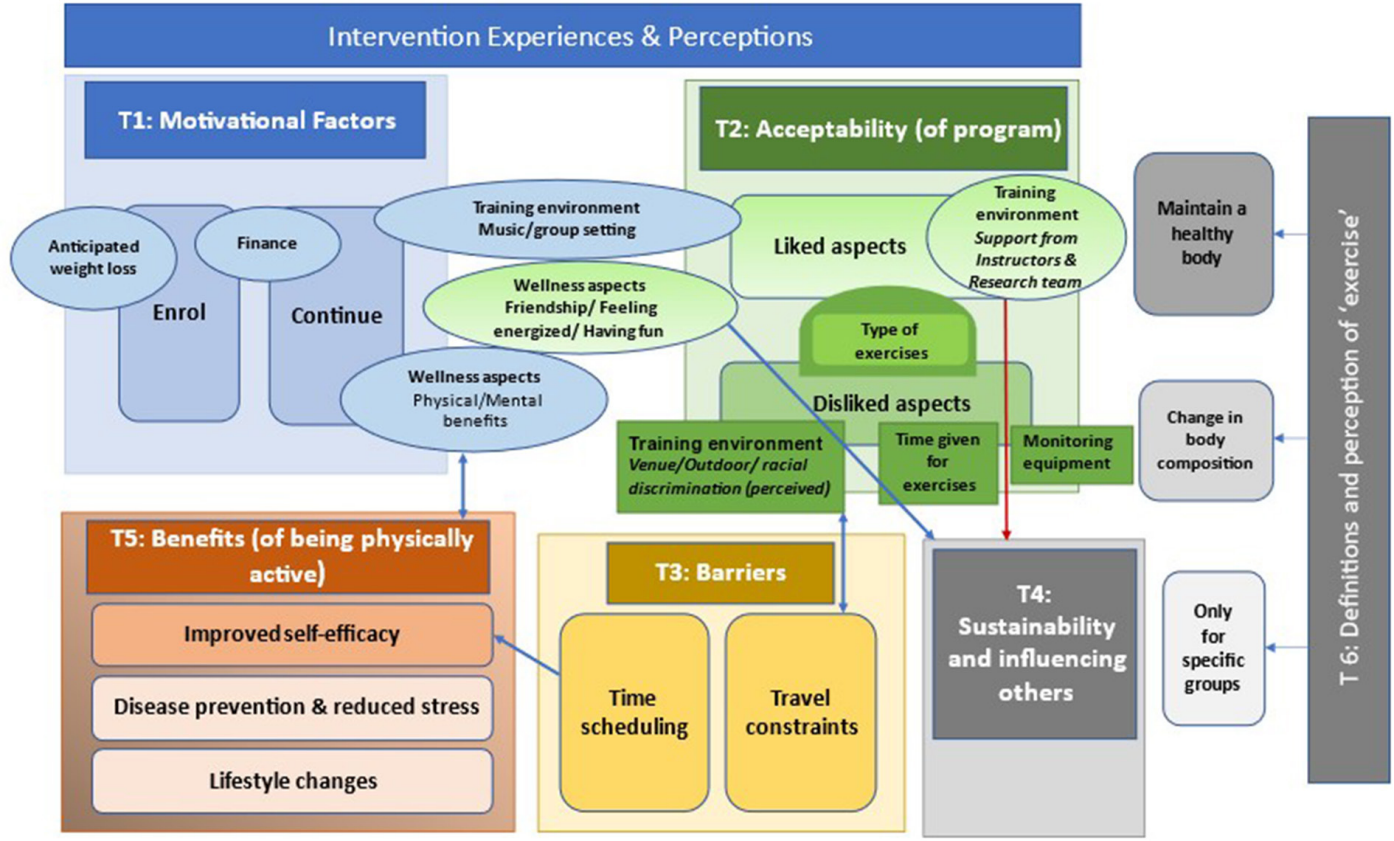
Themes and subthemes with possible associations and overlaps.

Figure 2 presents these themes as described in the following presentation of the results and shows some of the possible associations and overlaps between the various themes and sub-themes.

#### Theme 1: Motivational factors

During the analysis process, factors that the participants perceived to play a motivational role during the intervention were grouped together into two sub-themes namely (1) those factors that motivated the participants to enroll in the intervention, and (2) factors that kept the participants in the intervention. These identified factors are to be distinguished from the aspects that participants liked about the intervention which are included in the next theme (acceptability of the programme). Although there are overlaps, the codes informing this theme were drawn specifically from discussion points around what motivated the participants to enroll and then stay in the intervention.

##### Motivation to enroll

Extrinsic factors played an important role in the decision of participants to enroll in the intervention. Perceived financial advantage was mentioned by participants in all FGDs and some IDIs. This was followed by anticipated weight loss with some participants indicating that both factors played a role in their decisions.

“*The money, because I was like, I saw the pamphlet, then I said okay this is so much money, then I was like I need money, and this is going to help me in many things that I need”*. (**IDI: Participant 36;** ***exercise*** ***participant***).

“*My personal goal was basically to lose weight. I wanted to see how much weight I could lose within those three months…….”* (**IDI: Participant 25;** ***exercise*** ***participant***).

“*This exercise sessions I was going to lose weight. I was going to be on good shape, and I was going to get money”* **(FGD 4: Participant 45;** ***control participant*)**.

The in-depth interviews revealed additional insight into why financial considerations were so important, with participants reporting that they bought basic food (often refined carbohydrates) with the money.

“*The hamper. The rice, the mealie meal, the flour and the sugar, white sugar”*. **(IDI: Participant 19;** ***exercise participant*)**.

##### Motivation to continue

Participants reported both extrinsic (financial, weight loss, training environment and support from others) and intrinsic (enjoyment and mental wellness) factors that motivated them to remain in the intervention. Financial considerations remained important even in the face of challenges to continue.

“*My time was also affected by the exercise sessions, especially the 4 o'clock one because I would end up getting home at about 7pm. I would arrive home late and that would affect my cooking schedule, and they would shout at me at home for being late, but I didn't give up because I would be getting money.”* **(FGD1: Participant 31;** ***exercise participant*)**.

The participants felt toned and fitter as they could now walk long distances faster and climb stairs without becoming breathless.

*But then I didn't really care about losing weight because I actually felt fitter, I can walk from …. [suburb of Cape Town] to here in 10 minutes. So those were one of the things that I felt the exercise sessions really helped me with”* **(IDI: Participant 23;** ***exercise participant*)**.

Influence of family and friends also served as motivators for participants from both the FGD's and IDI's.

“*I've got a lot of support from my family. They actually gave me the go ahead, if it is going to work for you, do it. We are behind you all the way. There was also a point where I changed the way I eat, so they were also supporting me in that and my friends also got support from them, there was no negativity”*. **(FGD2: Participant 29;** ***exercise*** ***participant*)**.

“*My family, they motivated me to go to the exercise because it is important for the health”*. **(IDI: Participant 36;** ***exercise participant*)**.

Other participants however reported remaining motivated despite the negative opinions of friends and family. They persevered with the intervention even though their families expressed that their weight was “normal;” receiving remarks that they cannot see that they have lost any weight; or were warned about the negative effects of losing weight.

“*My friends. My family was like no your body is okay, and we love you as you are. You don't have to lose weight; you know how family is. They were just so supportive of you that they don't even see that there is a problem……But what about me? What about what I want? So, I told myself that I am going to do it and I did it”*. **(FGD2: Participant 29;** ***exercise*** ***group*)**.

*No, people were like, are you really exercising we see no difference. They were demotivating me and it was annoying me because they say when you exercise it's like chemotherapy you just go and then come out very thin like thin, extremely thin. It doesn't work like that. With exercise, it takes time to show that you are losing weight.”*. **(FGD4: Participant 25;** ***control group*)**.

#### Theme 2: Acceptability of the programme

Participants shared their experiences about participating in the intervention and what they liked or disliked about the exercise programme, providing insight into the acceptability of the programme. The disliked aspect can be distinguished from the barrier theme (theme 3) in that the barriers are mostly logistical aspects that made it difficult for the participants to attend the sessions while the disliked aspects were related to various aspects of the exercise programme. Likes and dislikes were equally distributed with many of the aspects of the programme appearing in both the “like” and “dislike” sub-themes, such as assistance from trainers and the types of exercises.

##### Aspects that were liked

Four main aspects came to the fore when participants discussed what they liked about the intervention. Three of these relate to the training environment. Participants liked and appreciated the support and assistance from the trainers and research team, this was followed by the type of exercises, music as part of exercise routines and lastly, wellness aspects (including friendship, feeling energized and having fun).

Positive experiences around support from the intervention team included factors such as having patience with and appreciating their challenges such as being late for the exercise classes.

“*The things that I loved most about the exercise is the aerobic sessions, the fact that when we are tired, instructors would be patient with us. They would be very supportive*. **(FGD1: Participant 24;** ***exercise participant*)**.

As highlighted in the previous quote, aerobics was the type of exercise session that was enjoyed the most. Music was used with all exercise routines and this component of the exercise programme was enjoyed by many participants. The importance of music was reiterated in the IDIs.

In addition, participants enjoyed training in a group setting and making new friends. This was reaffirmed in the IDIs.

“*The one thing that I like the most is that we do it as a group, firstly. It's not like you're doing it alone. I can do it with someone else, with people, that is nice and different types of exercises that I enjoyed is that what the question was about”* **(FGD3: Participant 23;** ***control group*)**.

##### Aspects that were disliked

Five aspects of the intervention were identified by the participants as contributing to negative experiences. The most discussed were the training environment followed by the types of exercises, time allowed for the exercises, the physical activity monitoring equipment and lastly, the instructors.

The venue, outdoor exercises and perceived racial discrimination were factors in the training environment that contributed to negative experiences. Concerning the racial discrimination some participants indicated that they sometimes felt unwelcome in the training venue and perceived this to be because they were black.

“*I feel like sometimes when we are here, other people down there, were looking at us badly because we are black, they used to say that we are disturbing them, especially when there was another class. They would want us to put our music softer but when we were outside, no one would complain”*. **(FGD1: Participant 19;** ***exercise participant*)**.

Participants identified that the heart rate monitors were uncomfortable to wear. Some of the participants also disliked certain high intensity exercises such as running up and down stairs, sit-ups, jumping jacks and running outside in the sun.

“*Having to wear equipment while exercising. Not the watch. Our equipment is very irritating. I get irritated by just looking at it”* **(FGD 3: Participant 44;** ***exercise participant*)**.

Time available to complete the exercise sessions and negative experiences with the instructors were also raised as factors that negatively affected experiences, the latter were raised by a participant during the IDIs.

“*I think it would be a bit more fun if the starter could maybe try to have different trainers with different experience… If there were different trainers maybe that would make things funnier or exciting. There were times when she will seem like she was not in the mood for exercise sessions”*. **(IDI: Participant 23;** ***exercise participant*)**.

#### Theme 3: Barriers

The most common barriers were travel constraints and lack of time. Some participants felt that the venue was not central, and they had to travel long distances to get to the venue. Three of the five participants mentioned the issue of transport as a barrier during the IDIs.

“*It does not accommodate everyone, only accommodate those who are closer to the buildings…. So, the traveling is also a barrier”* **(FGD2: Participant 29;** ***exercise participant*)**.

Most of the participants identifying time as an issue were university students, some of whom worked part time in addition to their studies and had challenges finding time to attend the programme.

“*It affected my daily life when I had things to do, and I had to come here to the gym………So it was very difficult for me to manage time because I had to come here at half past four. I was always late”*. **(FGD1: Participant 27;** ***exercise participant*)**.

#### Theme 4: Sustainability and influencing others

Participants shared their perceived enthusiasm to continue with the exercise programme after the end of the intervention and influence others to exercise. All FGDs participants indicated that they would encourage family and friends to start exercising. Further, being more physically active as a result of the intervention made them more aware of the benefits and importance of exercise and physical activity.

“*I will say yes or motivate someone to go to the gym because black people's mentality is that- one has to have curves. We are the ones, we are Black, you understand because when you're exercising like you are minimizing certain illness so I would say yes”* **(FGD4: Participant 32;** ***control participant*)**.

Continuing with an exercise programme came up in the FGDs and was explored further in the IDIs. Three of the five participants who participated in the IDIs continued training on their own after the intervention, one with the help of a volunteer instructor in an informal group setting.

“*I jog with my friends to the certain place and then when we get there we meet like other people and there's like [Xhosa] we have an instructor, but we don't pay. There is an instructor, but they do it for fun, for the love of it”*. (**FGD1: Participant 19;** ***exercise*** ***participant*)**.

One participant however reported difficulty in being consistent with the exercise regime mostly because of a lack of a supportive training environment.

“*Because I don't have a personal trainer, so I don't have someone looking after me. I do go sometimes, sometimes I don't go”*. **(IDI: Participant 23;** ***exercise participant*)**.

#### Theme 5: Benefits of being physically active

Most participants acknowledged that after completing the programme they were more aware of the benefits and importance of being physically active. The perceived benefits could be grouped in three sub-themes. Improved self-efficacy was the strongest sub-theme, followed by disease prevention and reduced stress levels, and lastly, lifestyle changes.

Some participants reported improved self-efficacy in the form of coping and scheduling skills. They expressed that the exercise programme helped improve their ability to cope with busy schedules and assisted with scheduling their busy daily routines.

“*Third year students have a lot of work so if I come home late, my body is tired but since I have been exercising, I have been coping. I would leave my room at 4 or 5 and I would have to come back and cook, eat and do schoolwork but what I realized was that I could cope unlike before when I used to sleep for 30 minutes. Since the gym I feel alright so that was the positive part about the gym. I could cope and be able to climb the stairs”*. **(FGD4: Participant 37;** ***control participant*)**.

“*So, like the fact that I had to allocate like some of my time to the gym, like to commit some of my time to this gym thing that's happening, it helps me to plan my daily activities”*. **(FGD1: Participant 24;** ***exercise group*)**.

Participants perceived that exercise increased their energy levels, reduced stress and may reduce risk of diseases such as high blood pressure, diabetes and heart disease.

“*like I see exercising as a tool that reduces high blood pressure”*. **(FGD4: Participant 32;** ***control*** ***participant*)**.

“*Exercise for me personally is a stress reliever”*. **(FGD3: Participant 39;** ***exercise*** ***participant*)**.

The participants furthermore perceived exercise to be associated with positive lifestyle changes such as changes in diet and participating in more active leisure time activities (i.e., going to the gym).

#### Theme 6: Definitions and perceptions of “exercise”

Several sub-themes emerged from the discussion around what the term “exercise” means. Participants defined “exercise” most often in terms of maintaining a healthy body, followed by changes in body composition such as weight loss and increased muscle mass, engaging in any physical activities, and lastly the perception that it is something that a specific group of people do.

“*I think the word exercise means getting yourself to be healthy, trying to change your body, get more muscles by doing exercises”*. **(FGD3: Participant 39;** ***exercise*** ***participant*)**.

“*I feel like exercise has to do more with physical activities than anything else. It is a physical activity. You don't have to do a certain thing and say it's exercising. You can walk, you can [do] anything, moving your body in general”*. **(FGD3: Participant 44;** ***exercise*** ***participant*)**.

A few of the participants from the FGD perceived exercise to be associated with ethnicity.

“*like I'm going to be very honest, like the word exercise to me, like it has the whiteness on it, like this whiteness, like it's for white people”*. **(FGD1: Participant 24;** ***exercise participant*)**.

## Discussion

In this study we aimed to gain an understanding of the experiences and perceptions of young Black SA women living with obesity following their participation in a 12-week structured exercise intervention. Six themes were identified from the focus groups and in-depth interviews and included motivating factors, acceptability of the programme, barriers, sustainability and influencing others, benefits of being physically active, and perceptions of the term “exercise”.

Financial advantage in the form of remuneration for costs relating to travel and time were identified as an important extrinsic motivator for enrolling and staying in the programme. This is not a unique finding as financial incentives have, even in high income settings, shown to be effective in behavior change and achieving physical activity goals, as well as to moderate the effects of self-efficacy on weight-loss in adults with obesity participating in interventions (21, 22). Although there is no data on the role of financial gain on physical activity interventions within the South African context, conditional cash transfers have been used in South African high school girls to reduce HIV incidence among young women and address structural factors such as poverty (23). However, the amount of money provided to our participants as reimbursement for their travel and time was small and the perception of financial advantage gained from the project may point to the socio-economic hardships suffered in the communities from which our participants were drawn (24). This opinion is supported by the finding that participants reported using the money to buy high-energy dense staple foods such as maize-meal and sugar. These choices suggest high levels of food insecurity in the households of our participants. The consumption of high energy dense foods in low socio-economic populations and the link with obesity has been consistently reported in the literature (25, 26).

In addition to the financial incentive, participants reported weight loss (anticipated and actual) as a motivator for enrolling and staying in the intervention. Participants were reminded at recruitment that substantial changes in body weight are not commonly shown in response to exercise only interventions (27, 28). Although only a small but significant weight loss was achieved by the intervention group (~1 kg) (20), this was experienced positively by the participants. Physical benefits such as a perceived increase in fitness levels and muscle tone could have contributed to these positive experiences. The multiple socio-cultural, environmental and behavioral factors involved in the risk for Black SA women to become obese (25) provides a further understanding for why even a small weight loss could have been considered an achievement.

Another benefit reported by participants was an improvement in psychological well-being (improved mood and self-esteem). These findings are supported by quantitative results previously reported from the same intervention, showing reduced symptoms of depression and improved sleep quality in response to the exercise training (29). Our findings are also consistent with previous research findings which suggested that improved psychological and physical well-being are related to increased exercise adherence in women with obesity (30). A study by Cleo et al. (31) reported that participants in a weight loss programme were impressed by the “indirect ripple effect” of health benefits they experienced aside from weight loss, and this seems to be true for our study as well.

Another benefit identified is the perceived increased level of self-efficacy of participants. Some indicated that participation in the programme assisted them with improving their scheduling behavior while feelings of enhanced physical fitness, reduction of stress, and improved mood were all evident and have previously been shown to strengthen self-efficacy (32). An increase in self-efficacy is furthermore associated with persistence in exercise training even in the presence of challenging barriers (30). Barriers to attending the training sessions in our study included lack of time, distance to the training venue, and limited transport to the training venue, all of which have been previously reported in low socioeconomic settings (14). Moreover, participants reported aspects of the programme that they did not like such as the venue, some personal characteristics of the trainers, high intensity exercises and perceived racial discrimination at the venue. The latter is important as racial “everyday discrimination” were found to be positively related to distress (33) and potentially may weaken the positive experiences of participants to similar programmes. When considered together with the perception of exercise as an activity done by “white” people only, careful attention should be given to this aspect in exercise programme development. The dislike of high intensity exercise supports the results of Foster et al. (34) that in a group of relatively untrained young adults the exercise regime with the highest intensity had the lowest enjoyment score.

Intrinsic and extrinsic aspects of the training programme and training environment, such as support by the instructors and group, types of exercises, friendships, fun and music were identified as well-liked aspects of the programme. Participants suggested that these positively experienced factors are important when considering the sustainability of the programme, made them feel motivated to influence others to exercise and motivated them to continue with the intervention. According to Withall et al. (14) once people are active, high levels of social interaction, interest and enjoyment, are associated with improved levels of retention. Our results show that the perceived benefits (motivators) outweighed dislikes and barriers and facilitated the continuation and completion of the intervention. Not only did most participants (87%) complete the programme, but some went on to continue exercising, and all saw themselves as potentially motivating loved ones to embark on exercise programmes.

A limitation of the study is that no member checking was done to explore the credibility of the findings. Various aspects of the study however contribute to the credibility of the findings. Firstly, the study was preceded by formative work in a similar group of women and the research team had insight into the lived experiences of the study participants. Although the exercise training was prescriptive and not self-selected, the mode of training was based on this formative work (6). Furthermore, extensive notes were taken by a scribe and these notes were considered during the analysis process. The current study only included participants that completed the intervention, as drop out participants were not contactable, but the aim was to describe the perceptions and experiences of those that completed the intervention.

## Conclusion

The current study showed that the young Black SA women from a low socioeconomic setting that participated in a 12-week structured exercise intervention identified perceived financial gain and perceived weight loss as motivators for exercise adherence. Furthermore, adherence to an exercise training programme was identified to enhance mood, increase self-esteem, self-efficacy, and the development of skills to cope with perceived barriers. A targeted intervention approach that incorporates elements such as the presence of an exercise instructor, music and dancing in a group setting should be considered when developing public health programmes for sustainability in young women from low-income settings. Lastly, ease and cost of access to training facilities is key to increasing and/or maintaining participation adherence in similar exercise training programmes in this population and should be considered for programme sustainability. Collectively, improved self-efficacy may play an important role in supporting exercise behavior and should be a component that is assessed to ensure sustainability when implementing future exercise programmes. We are of the opinion that financial incentives are not likely to be a sustainable approach for interventions. Further research is required to address other sustainable motivators to exercise adherence such as self-selected exercise training modes and intensities, and combining diet and exercise to maximize weight loss.

## Data availability statement

The raw data supporting the conclusions of this article will be made available by the authors, without undue reservation.

## Ethics statement

The study was approved by the Human Research Ethics Committee at the University of Cape Town (HREC REF: 054/2015). The patients/participants provided their written informed consent to participate in this study.

## Author contributions

JG, AM, and LM conceived and designed the study and were involved in data analysis. LP analyzed the transcripts, generated codes, and collated codes into potential themes and sub-themes. AV cross-validated the coding. LP prepared figures and drafted the manuscript. AV, JG, AM, and LM read and edited the manuscript and approved the final version. All authors contributed to the article and approved the submitted version.

## Funding

This study was funded by the National Research Foundation of South Africa (NRF), Competitive Programme for Rated Researchers (Grant No: 93577).

## Conflict of interest

The authors declare that the research was conducted in the absence of any commercial or financial relationships that could be construed as a potential conflict of interest.

## Publisher's note

All claims expressed in this article are solely those of the authors and do not necessarily represent those of their affiliated organizations, or those of the publisher, the editors and the reviewers. Any product that may be evaluated in this article, or claim that may be made by its manufacturer, is not guaranteed or endorsed by the publisher.

## Abbreviations

AIDS: Acquired Immune Deficiency Syndrome
ANOVA: Analyses of variance
BMI: Body Mass Index
FGDs: Focus group discussions
GCP: Good Clinical Practice
HR: Heart rate
HIV: Human Immunodeficiency Virus
HREC: Human Research Ethics Committee
IDIs: In-depth interviews
ICH: International Conference on Harmonization
HR_max_: Maximum heart rate
%HR_max_: Percentage of maximum heart rate
SD: Standard deviations
SA: South Africa
ZAR: South African Rands
ST: Subthemes
T: Themes.
